# Supplementary material for: Multiple objective immune wolf colony algorithm for solving time-cost-quality trade-off problem
Source: PLoS One. 2023 Feb 9;18(2):e0278634. doi: 10.1371/journal.pone.0278634 (PMC9910742; doi:10.1371/journal.pone.0278634)

Project	Start Data	End Date	Quantities	Duration
Construction preparation	2008-3-1	2008-8-31	1318km	6
Beam making	2008-5-1	2010-1-31	1060.6km	21
Beam erection	2008-12-1	2010-7-31	30984hole	20
Tunnel engineering	2008-5-1	2010-2-28	16.08km	22
Subgrade engineering	2008-5-1	2010-8-31	241.3km	28
Yellow River Bridge	2008-4-1	2010-6-30	5.14km	27
Ballastless track	2009-10-1	2010-10-31	1299km	13
Turnout/ track laying/ Whole track	2010-7-1	2011-4-30	3126.9km	10
Zaobeng pilot section	2008-3-1	2010-11-30	220km	33
Four electric engineering	2010-9-1	2011-4-30	1318km	8
Station house	2009-5-1	2011-4-30	896000m²	25
Joint commissioning	2011-5-1	2011-7-31	1318km	3
Railway operation	2011-8-1		1318km	

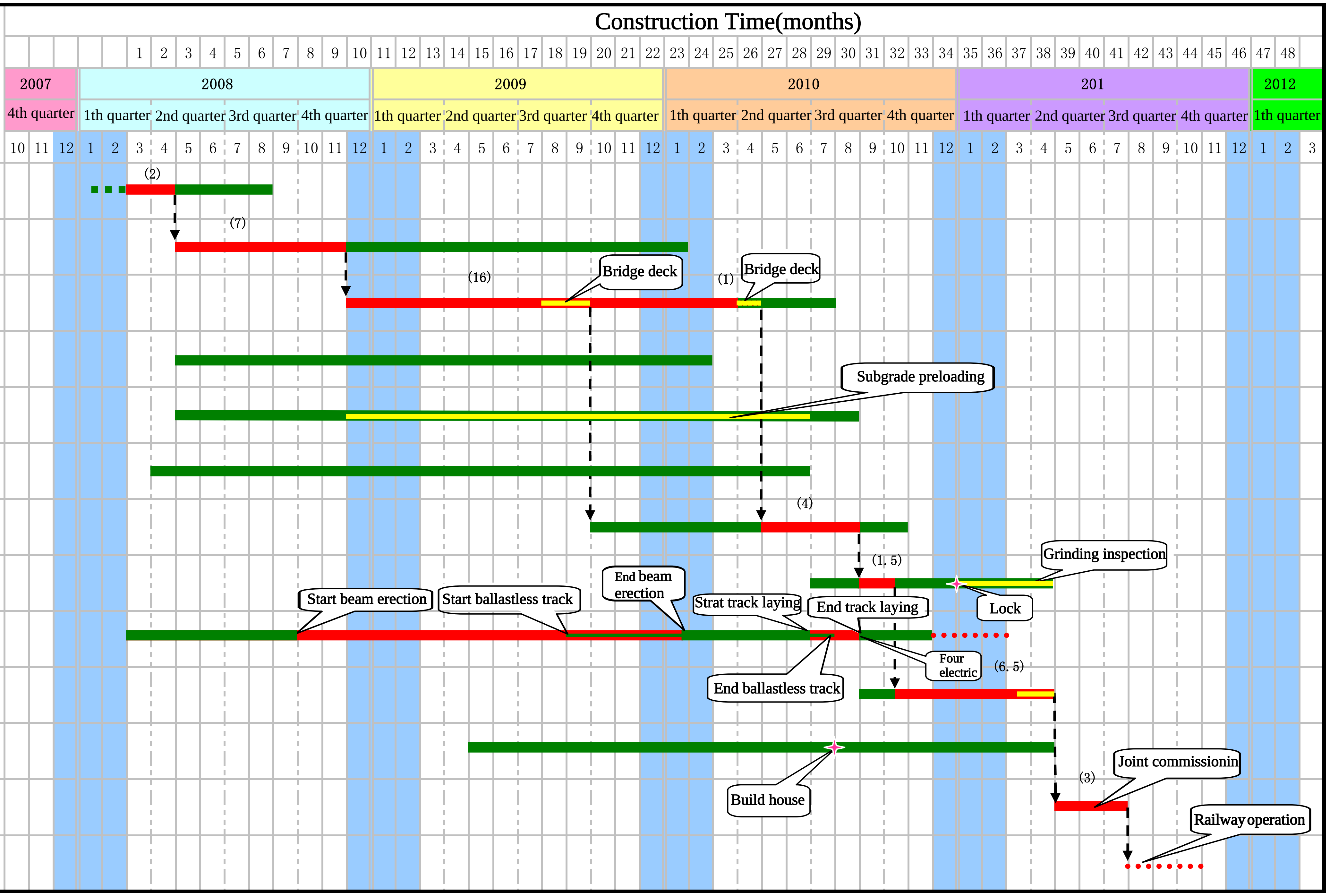

Supplement: S1 File — (PDF) [file pone.0278634.s001.pdf]
