# Supplementary material for: Multiple objective immune wolf colony algorithm for solving time-cost-quality trade-off problem
Source: PLoS One. 2023 Feb 9;18(2):e0278634. doi: 10.1371/journal.pone.0278634 (PMC9910742; doi:10.1371/journal.pone.0278634)
Supplement: Attachment — Submitted filename: Response to Reviewers.docx [file pone.0278634.s002.docx]

**Changes made to the Paper**

**Dear Editor**,

The authors gratefully acknowledge the constructive comments on the paper entitled “**Multiple objective immune wolf colony algorithm for solving time-cost-quality trade-off problem**” (Manuscript Number: PONE-D-22-22367), offered by the editor and the anonymous referees. We have reviewed the comments and generally agree with those. Accordingly, we have made changes in the paper in line with the referee’s observations to the extent practicable. We take this opportunity to thank each of the individuals involved in the process. We express our sincere gratitude for their in-depth reviews which have helped improve the quality of the paper significantly.

Please find below our response to the comments in the order in which they have been raised.

**Reviewer #1:**

**Comment to the Authors**

1. In the introduction, the authors provide reviews on metaheuristics and multi-objective optimization. However, what are the drawbacks of current research? Please provide the motivation of the manuscript. Besides, what are the contributions of this manuscript?

**Response**

Thank you for your suggestion. As suggested by reviewer, we have added the suggested content to the manuscript **(Page7, lines 131-145)**. The details are as follows:

It is not difficult to conclude from the above literature review that TCQT has been widely addressed and effectively resolved. However, the existing TCQT mathematical model has not considered the impact of reward and punishment factors on construction projects, and intelligent optimization algorithms with better solution performance need to be proposed. Thereby, the contributions of this study are stated as follows: (1) clarify the nonlinear relationship between time and bonus-penalty cost, a new bonus-penalty cost model is presented. Therefore，a new multi-objective mathematical model of the time-cost quality trade-off problem with several equality and inequality constraints is established. (2) A new multi-objective immune wolf swarm algorithm has been developed to solve the trade-off problem of time, cost, and quality. The proposed MOIWCA can solve the problem that the traditional wolf colony algorithm is easy to fall into a local optimal solution. At the same time, in order to improve the search speed and optimization performance of the algorithm, the cross operation and immune operation in the immune algorithm are improved. The algorithm can provide a representative and managed Pareto set for the time-cost-quality trade off problem. (3) To make sure that the proposed MOIWCA works and can be used, we use a high-speed railway construction project as a case study to show that the proposed method is better.

2. The author lacks some state-of-the-art algorithms. For research on multi-objective algorithms, the compared algorithms in this manuscript are limited. Please add some state-of-the-art algorithms, e.g., NSGA-II, MOEA/D, MOSPO, MOAOS, MOAVOVA, and some multi-objective optimization methods.

**Response**

Thank you for your suggestion. As suggested by reviewer, we have added NSGA-II and MOEA/D, to further strengthen the comparison and analysis between different algorithms in the manuscript **(Pages 26-27, lines 524-525 and 533-534)**. The details are as follows:

**Table 1.** Comparison of Test Results of Rosenbrock Function Using IWCA, IGPSO, NSGA-II and MOEA/D

|  | Average running time (ms) | Average iteration generations | Mean error | Standard deviation | Success rate (%) | Best minimum value |
| --- | --- | --- | --- | --- | --- | --- |
| IGPSO | 8,687 | 2,183 | 0.429618 | 0.256765 | 100.00 | 0.007728 |
| IWCA | 7,568 | 1,750 | 0.415738 | 0.235836 | 100.00 | 0.004258 |
| NSGA-II | 15,366 | 3,628 | 0.820524 | 0.446531 | 100.00 | 0.008795 |
| MOEA/D | 11,082 | 2,825 | 0.646358 | 0.315873 | 100.00 | 0.007936 |

**Table 2.** Comparison of Test Results of Rastrigin Function Using IWCA, IGPSO, NSGA-II and MOEA/D

|  | Average running time (ms) | Average iteration generations | Mean error | Standard deviation | Success rate (%) | Best minimum value |
| --- | --- | --- | --- | --- | --- | --- |
| IGPSO | 18,046 | 3,928 | 0.367794 | 0.241865 | 100.00 | 0.004157 |
| IWCA | 9,658 | 2,486 | 0.285682 | 0.224783 | 100.00 | 0.003265 |
| NSGA-II | 27,863 | 6,838 | 0.824563 | 0.468735 | 100.00 | 0.013648 |
| MOEA/D | 22,648 | 5,082 | 0.693425 | 0.336749 | 100.00 | 0.008543 |

3. Please use more powerful criteria such as HV or Delta. GD and IGD are basically the same.

**Response**

Thank you for your suggestion. As suggested by reviewer, we select SP and HV for analysis in the manuscript **(Pages 31-33, lines 614-652)**. The details are as follows：

We compared MOIWCA performance against NSGA-II, MOPSO and MODE to assess comparative effectiveness. For comparison purposes, all four algorithms used an equal number of function evaluations, had a population size of 300, and a maximum of 500 generations. In NSGA-II, the crossover probability is set at 0.5 and the mutation probability is set at 0.1. In MOPSO, the two learning factors *c_1_*, *c_2_* are both chosen at 1.5, and the inertia factor *w* is set in the range of 0.2–0.6. The MOIWCA control parameters remained the same, as stated previously in Table 3. Thirty independent runs are carried out for all experiments.

Multi-objective optimization problem performance measures are more complex than those of single-objective optimization problems. Three issues are typically considered: (1) convergence to the Pareto optimal set; (2) preservation of diversity in Pareto optimal set solutions; and (3) the maximal distribution bound of the Pareto optimal set [38]. In the literature, the researchers have suggested numerous quality indicators. In this paper, the Spacing metric, and the Hyper volume (HV) metric are selected to evaluate the performance of different algorithms.

(1) Spacing metric: This indicator assesses the degree of distribution of non-dominated solutions. The mathematical definition of the spacing metric may be given as:

$\Delta=\frac{\sum_{i=1}^{m} d\left( E_{i},\Omega\right)+\sum_{X\in\Omega} \left| d\left( X,\Omega\right)-\bar{d} \right|}{\sum_{i=1}^{m} d\left( E_{i},\Omega\right)+(\left| \Omega\right|-m)\bar{d}}$	(40)

where $\Omega$is a set of non-dominated Pareto-front solutions, ($E_{1},E_{2},\ldots,E_{k})$ are *k* extreme solutions in the set is true Pareto-front, *m* is the number of objectives and $d\left( X,\Omega\right)={min \atop Y\in\Omega, Y\neq X}\left\| F\left( X \right)-F(Y) \right\|$ is the minimum Euclidean distance between solution X and its neighboring solutions in the obtained non-dominated X set, $\bar{d}=\frac{1}{\left| \Omega\right|}\sum_{X\in\Omega} d\left( X,\Omega\right)$ is the mean value of all $d\left( X,\Omega\right)$, $\left| \Omega\right|$ is the total solutions in $\Omega$ set. A value of 0 indicates that all members of Pareto optimal solution are equally spaced. The smaller the value, the better distribution and diversity of non-dominated solutions. Table 6 shows a comparison of the Spacing metric for different algorithms.

**Table 6. Comparison of Spacing metric for different algorithm**

|  | MOIWCA | MODE | MOPSO | NSGA-II |
| --- | --- | --- | --- | --- |
| Best | 0.4336 | 0.4453 | 0.4627 | 0.4852 |
| Worst | 0.6132 | 0.7025 | 0.9416 | 0.9653 |
| Average | 0.5235 | 0.5431 | 0.7123 | 0.7356 |
| Standard Deviation | 0.0397 | 0.0586 | 0.1542 | 0.1653 |

(2) Hyper-volume (HV): This indicator calculates the volume (in the objective space) covered by members of a non-dominated set of solutions $\Omega$ to a problem that works to minimize all objectives. A hypercube $v_{i}$is constructed for each solution $X_{i}\in\Omega$, with reference point *W* and the solution *X_i_* as the diagonal corners of the hypercube. The reference point may be found simply by constructing a vector of the worst objective function values. Thereafter, a union of all hypercubes is found, with the HV of this union calculated as:

$HV=\bigcup_{i=1}^{\left| \Omega\right|} v_{i}$	(41)

Algorithms with larger HV values are desirable. The HV value of a set of solutions is normalized using a reference set of Pareto optimal solutions with the same reference point. After normalization, the HV values are confined to range [0,1]. Table 7 lists the results for each of the four compared algorithms in terms of HV. From Table 7, we can see that the proposed model gets the highest HV values. This means that the MOIWCA has better convergence and diversity performance than the other algorithms.

**Table 7. Comparison of HV metric for different algorithm**

|  | MOIWCA | MODE | MOPSO | NSGA-II |
| --- | --- | --- | --- | --- |
| Best | 0.9486 | 0.8965 | 0.9284 | 0.8652 |
| Worst | 0.7164 | 0.6932 | 0.4683 | 0.2473 |
| Average | 0.8207 | 0.7648 | 0.7136 | 0.5241 |
| Standard Deviation | 0.0519 | 0.0546 | 0.1483 | 0.2062 |

4. Please review the paper for proper use of the English language.

**Response**

Thank you for your careful review. The manuscript has been thoroughly revised and rewritten by a native English speaker.

5. Please update ref with following:

Khodadadi, N., Azizi, M., Talatahari, S., & Sareh, P. (2021). Multi-objective crystal structure algorithm (MOCryStAl): introduction and performance evaluation. IEEE Access, 9, 117795-117812.

Azizi, M., Talatahari, S., Khodadadi, N., & Sareh, P. (2022). Multi-Objective Atomic Orbital Search (MOAOS) for Global and Engineering Design Optimization. IEEE Access.

Khodadadi, N., Abualigah, L., & Mirjalili, S. (2022). Multi-objective Stochastic Paint Optimizer (MOSPO). Neural Computing and Applications, 1-24.

**Response**

We are extremely grateful to you for pointing out this problem. The references you provided are very consistent with the research content, and have high academic value, which is very helpful to our paper. Therefore, we decided to use the three articles you suggested to further enrich our references in the manuscript **(Pages 5-6, lines 113-117 and 122-130)**. The details are as follows：

In Khodadadi N, Azizi M, Talatahari S, Sareh P [25] considering the multi-objective optimization problem of multiple performance indicators inspired by the principle of crystal structure formation, a meta heuristic algorithm called the Crystal Structure algorithm (Crystal) is proposed, and the algorithm is evaluated. The results show that the algorithm provides excellent results when dealing with multi-objective problems. Based on Azizi M, et al [27], Multi-Objective Atomic Orbital Search (MOAOS) is proposed to solve multi-objective optimization problems. By using MOAOS to evaluate benchmark problems ZDT and DTLZ, the results show that this algorithm can produce superior or similar results compared with other meta-heuristic methods. Khodadadi N, Abualigah L, Mirjalili S [28] made appropriate changes to the stochastic paint optimizer (SPO) and proposed a multi-objective stochastic paint optimizer (MOSPO) to solve multi-objective optimization problems. Compared with MOPSO MSSA and MOALO, MOSPO had high convergence and excellent Pareto front results in dealing with multi-objective engineering problems.

**Reviewer #2:**

**Comment to the Authors**

1. The readability and presentation of the study should be further improved. The paper suffers from language problems. It is suggested to proofread by a native speaker or a proofreading agent.

**Response**

Thank you for your careful review. The manuscript has been thoroughly revised and rewritten by a native English speaker.

2. The importance of this study is not clear. The authors are suggested to state the reason for proposing this algorithm.

**Response**

Thank you for your suggestions. As suggested by reviewer, we add the importance of this paper and the reason for the proposed algorithm in the manuscript **(Page 6, lines 131-134)**. The details are as follows:

It is not difficult to conclude from the above literature review that TCQT has been widely addressed and effectively resolved. However, the existing TCQT mathematical model has not considered the impact of reward and punishment factors on construction projects, and intelligent optimization algorithms with better solution performance need to be proposed.

3. It is recommended to revise the contribution of this study by highlighting its novelty and finding of this study.

**Response**

Thank you for your suggestions. As suggested by reviewer, we have revised the contributions of this study in the manuscript **(Page 6, lines 134-145)**. Our reply is as follows:

Thereby, the contributions of this study are stated as follows: (1) clarify the nonlinear relationship between time and bonus-penalty cost, a new bonus-penalty cost model is presented. Therefore，a new multi-objective mathematical model of the time-cost quality trade-off problem with several equality and inequality constraints is established. (2) A new multi-objective immune wolf swarm algorithm has been developed to solve the trade-off problem of time, cost, and quality. The proposed MOIWCA can solve the problem that the traditional wolf colony algorithm is easy to fall into a local optimal solution. At the same time, in order to improve the search speed and optimization performance of the algorithm, the cross operation and immune operation in the immune algorithm are improved. The algorithm can provide a representative and managed Pareto set for the time-cost-quality trade off problem. (3) To make sure that the proposed MOIWCA works and can be used, we use a high-speed railway construction project as a case study to show that the proposed method is better.

4. This study suffers from a lack of deep literature review on new and improved metaheuristic algorithms such as Migration-based moth-flame optimization algorithm, Hybridizing of whale and moth-flame optimization algorithms to solve diverse scales of optimal power flow problem, An improved moth-flame optimization algorithm with adaptation mechanism to solve numerical and mechanical engineering problems, and GGWO: Gaze cues learning-based grey wolf optimizer and its applications for solving engineering problems.

**Response**

We are extremely grateful to you for pointing out this problem. The references you provided are very consistent with the research content, and have high academic value, which is very helpful to our paper. Therefore, we decided to use the two articles you suggested to further enrich our references in the manuscript **(Page 5, lines 109-113 and 117-122)**. The details are as follows：

Nadimi-Shahraki, et al [24] proposed an improved moth-flame optimization (I-MFO) algorithm, introduced the adapted wandering around search (AWAS) strategy to escape the local optimal solution, evaluated the performance of the proposed algorithm through a benchmark function, and compared it with other well-known metaheuristic algorithms. Finally, I-MFO is used to solve practical mechanical engineering problems. To Nadimi-Shahraki MH, et al [26] reduce the high selection pressure and low diversification of GWO algorithm, a grey wolf optimizer based on gaze cue learning (GGWO) is proposed. The algorithm introduced two strategies: neighbor gaze cues learning (NGCL) and random gaze cues learning (RGCL)to enhance the optimization ability. The GGWO algorithm is compared with other algorithms. The results show that the GGWO algorithm has better performance.

5. It is recommended to consider the flowchart or pseudocode of the proposed algorithm.

**Response**

Thank you for your suggestions. In the paper, we have provided the flow chart of the proposed algorithm, that is, Fig 8. (Flowchart of MOIWCA for the fuzzy TCQT problem) **(Page 25, line 500)**.

Fig 8 Flowchart of MOIWCA for the fuzzy TCQT problem

6. It is recommended to experimentally report the weak points of the immune wolf colony algorithm. then clarify how the proposed algorithm can overcome these weak points to solve the time-cost-quality trade-off problem.

**Response**

Thank you for your suggestions. As suggested by reviewer, in order to better illustrate the advantages of the algorithm in dealing with the time cost quality tradeoff problem, we modified and supplemented the algorithm design part of the paper **(Pages 19-21, lines 392-416)**. The details are as follows:

The immune wolf colony hybrid algorithm proposed in this paper focuses on the combination of wolf colony algorithm and immune algorithm, which makes the hybrid algorithm have the advantages of immune algorithm's excellent solving performance in combinatorial optimization and wolf colony algorithm's fast convergence in solving problems, and avoids the disadvantages of immune algorithm's slow convergence speed in optimization problems and wolf colony algorithm's easy falling into local extremum. Therefore, the immune wolf colony hybrid algorithm provides a new way to solve the time-cost quality trade-off (TCQT) problem.

When using the immune wolf colony hybrid algorithm to solve the TCQT problem, this paper takes the individual wolf colony as the antibody of the immune algorithm, the odor concentration of prey as the antigen of the immune algorithm, and the odor concentration of the individual wolf colony as the fitness value of the current solution. The process of wolf colony searching, and trapping prey is to use immune wolf colony hybrid algorithm to solve the TCQT problem iteratively. In the process of wolf pack updating, it is always hoped that the wolf with high adaptability will be left behind. However, if the superior wolf is too concentrated, it is difficult to ensure the diversity of the whole wolf colony. Therefore, by using the mechanism of antibody concentration inhibition, the antibody with low affinity and high concentration will be suppressed, and the antibody with high affinity and low concentration will be retained and promote the production, to ensure the diversity of antibody groups.

The basic principle of IWCA is to combine the immune principle of antibody concentration inhibition mechanism and immune memory function in the immune algorithm with the wolf colony algorithm, calculate the concentration of wolves(antibody) and compare it with the initial wolf concentration (*C_u_*). If it is higher than *C_u_*, update the wolves using IWCA, otherwise, update the wolves using the self-adapting WCA. Self-adapting WCA can accelerate search efficiency. The immune operation increases wolf colony diversity, ensures convergence speed, and improves the global search ability and accuracy.

7. Equations 36 and 37 are not clear. Please clarify them.

**Response**

Thank you for your suggestions. We are sorry that we clearly did not express Equations 36 and 37. Equation 36 is based on equation 29, and uses the adaptive parameter adjustment in particle swarm optimization to improve rush factor $\theta$ **(Page23, lines 459-471)**. The details are as follows:

a. Perform dynamic self-adapting of calling behavior of the wolf colony. For solving the local optimal solution of WCA, by referring to the adaptive parameter adjustment method in the particle swarm optimization algorithm, change the rush factor $\theta$ in Eq. (29) into a dynamic weight coefficient$\theta^{*}$, to change the fixed step of the calling behavior. In this paper, dynamic adaptive calling behavior of WCA algorithm is proposed. The new calling behavior of the wolf colony according to the following equations:

$x_{id}^{k+1}=x_{id}^{k}+\theta^{*}\left( x_{ad}^{k}-x_{id}^{k} \right){step}_{b}$	(36)

$\theta^{*}=\left\{ \begin{aligned} \theta_{min}^{*}+\frac{\left( \theta_{max}-\theta_{min} \right)\left( F_{avg}^{k}-F_{min}^{k} \right)}{F_{id}^{k}-F_{min}^{k}} F_{id}^{k}\geq F_{avg}^{k} \\ \theta_{max}^{*} F_{id}^{k}<F_{avg}^{k} \end{aligned} \right.$	(37)

where $\theta^{*}$ is dynamic adaptive coefficient, $\theta_{max}^{*}$ and $\theta_{min}^{*}$ are the maximum and minimum inertial coefficients respectively; $F_{min}^{k}$ is the minimum value of fitness function of wolf colony in the *k* generation; $F_{id}^{k}$ is the value of fitness function of the wolf *i* in the *d* dimensional space when the *k* generation; $F_{avg}^{k}$ is the average value of fitness function

8. It is recommended to boost the visualization of this study by increasing the experimental evaluations.

**Response**

Thank you for your suggestions. We know that the supplement of experimental evaluations will improve the overall level of our paper, but the main purpose of the paper is to apply the proposed IWCA to solve the engineering problems of the Beijing Shanghai high-speed railway construction project, and the results show that the method is effective. However, we add the proposed IWCA comparison experiment between IGPSO, NSGA-II and MOEA/D. The comparison results are shown in Table 1 and Table 2. **(Pages 26-27, lines 513-541)** We will incorporate your suggestions into our future work, meanwhile, based on your suggestions, add performance comparison experiments with other intelligent algorithms to further evaluate the proposed algorithms in the future.

**Table 1.** Comparison of Test Results of Rosenbrock Function Using IWCA, IGPSO, NSGA-II and MOEA/D

|  | Average running time (ms) | Average iteration generations | Mean error | Standard deviation | Success rate (%) | Best minimum value |
| --- | --- | --- | --- | --- | --- | --- |
| IGPSO | 8,687 | 2,183 | 0.429618 | 0.256765 | 100.00 | 0.007728 |
| IWCA | 7,568 | 1,750 | 0.415738 | 0.235836 | 100.00 | 0.004258 |
| NSGA-II | 15,366 | 3,628 | 0.820524 | 0.446531 | 100.00 | 0.008795 |
| MOEA/D | 11,082 | 2,825 | 0.646358 | 0.315873 | 100.00 | 0.007936 |

**Table 2.** Comparison of Test Results of Rastrigin Function Using IWCA and IGPSO

|  | Average running time (ms) | Average iteration generations | Mean error | Standard deviation | Success rate (%) | Best minimum value |
| --- | --- | --- | --- | --- | --- | --- |
| IGPSO | 18,046 | 3,928 | 0.367794 | 0.241865 | 100.00 | 0.004157 |
| IWCA | 9,658 | 2,486 | 0.285682 | 0.224783 | 100.00 | 0.003265 |
| NSGA-II | 27,863 | 6,838 | 0.824563 | 0.468735 | 100 | 0.013648 |
| MOEA/D | 22,648 | 5,082 | 0.693425 | 0.336749 | 100 | 0.008543 |

9. It is recommended to determine the claims of this study and which experimental evaluation can support them.

**Response**

Thank you for your suggestions. We know that the supplement of experimental evaluations will improve the overall level of our paper, but the main purpose of the paper is to apply the proposed WOIWCA to solve the engineering problems of the Beijing Shanghai high-speed railway construction project, and the results show that the method is effective. As the response to question 8, through the comparative experiment with IGPSO, NSGA-II and MOEA/D, the proposed IWCA performs excellent in average running time, average iteration generations, mean error, standard deviation, success rate and best minimum value. We will incorporate your suggestions into our future work, meanwhile, based on your suggestions, add performance comparison experiments with other intelligent algorithms to further evaluate the proposed algorithms and determine the claims in the future.

10. The discussion section should be added in a more highlighting, argumentative way. The author should analyze the reason why the tested results are achieved.

**Response**

Thank you for your suggestions. As suggested by reviewer, not only we improved the analysis of Fig. 9, but also enriched the discussion and analysis of experimental results by adding Figs. 10-12. **(Pages 30-31, lines 593-613)**. The specific analysis contents are as follows:

The non-dominated solutions may also be used to optimize tradeoffs between any two objectives on a two-dimensional plane. Figs. 10-12 show the trade-off between time and cost, time and quality, and cost and quality, respectively. As illustrated in Fig 10, if the investment in the construction project is increased, the duration of completing the project will be shortened, and vice versa. As shown in the Fig 11, if the duration of the construction project is increased, the quality of the completed project will increase first and then decrease. The situation shows that excessive extension of the construction project will lead to a decline in the quality of the project. One of the possible reasons for the situation is that long hours of work will reduce the working efficiency of labors. As shown in the Fig 12, if the quality of construction projects is improved, the investment in construction projects will increase. However, blindly increasing investment will not make the quality higher and higher but can reduce the quality. One of the possible reasons for the situation is that the purpose of increasing costs is to shorten the duration of the construction project, and the reduction of time leads to the decline of the quality of the construction project.

Fig.10. Time-cost tradeoff analysis

Fig.11. Time-quality tradeoff analysis

Fig.12. Cost-quality tradeoff analysis

11. The single-point crossover operation based on the crossover of points with the same serial number is not clear. Please increase the understanding and impact of this operator in the proposed algorithm.

**Response**

Thank you for your suggestions. As suggested by reviewer, we re-describe the proposed single point crossover operation **(Page 23, lines 475-482)**. The details are as follows:

In this paper, a single point crossover operation based on the same gene number. The difference between the proposed method and the classical single point crossover operation is that the selection of crossover points is not random. The specific method is to randomly select two chromosomes, select the same gene number from the two parent chromosomes as the intersection point, and then split and exchange the remaining chromosomes on the right side from this point to obtain new offspring. The new crossover operation method can improve the running speed of the algorithm and ensure the diversity of the population. A single point crossover operation based on the same gene number is shown in Fig 6.

**Fig 6.** single point crossover operation based on the same gene number

12. The statistical evaluation analysis is recommended.

**Response**

Thank you for your suggestion. As suggested by reviewer, we select SP and HV for analysis **(Page 31-33, lines 615-652)**. The details are as follows：

We compared MOIWCA performance against NSGA-II, MOPSO and MODE to assess comparative effectiveness. For comparison purposes, all four algorithms used an equal number of function evaluations, had a population size of 300, and a maximum of 500 generations. In NSGA-II, the crossover probability is set at 0.5 and the mutation probability is set at 0.1. In MOPSO, the two learning factors *c_1_*, *c_2_* are both chosen at 1.5, and the inertia factor *w* is set in the range of 0.2–0.6. The MOIWCA control parameters remained the same, as stated previously in Table 3. Thirty independent runs are carried out for all experiments.

Multi-objective optimization problem performance measures are more complex than those of single-objective optimization problems. Three issues are typically considered: (1) convergence to the Pareto optimal set; (2) preservation of diversity in Pareto optimal set solutions; and (3) the maximal distribution bound of the Pareto optimal set [38]. In the literature, the researchers have suggested numerous quality indicators. In this paper, the Spacing metric, and the Hyper volume (HV) metric are selected to evaluate the performance of different algorithms.

(1) Spacing metric: This indicator assesses the degree of distribution of non-dominated solutions. The mathematical definition of the spacing metric may be given as:

$\Delta=\frac{\sum_{i=1}^{m} d\left( E_{i},\Omega\right)+\sum_{X\in\Omega} \left| d\left( X,\Omega\right)-\bar{d} \right|}{\sum_{i=1}^{m} d\left( E_{i},\Omega\right)+(\left| \Omega\right|-m)\bar{d}}$	(40)

where $\Omega$is a set of non-dominated Pareto-front solutions, ($E_{1},E_{2},\ldots,E_{k})$ are *k* extreme solutions in the set is true Pareto-front, *m* is the number of objectives and $d\left( X,\Omega\right)={min \atop Y\in\Omega, Y\neq X}\left\| F\left( X \right)-F(Y) \right\|$ is the minimum Euclidean distance between solution X and its neighboring solutions in the obtained non-dominated X set, $\bar{d}=\frac{1}{\left| \Omega\right|}\sum_{X\in\Omega} d\left( X,\Omega\right)$ is the mean value of all $d\left( X,\Omega\right)$, $\left| \Omega\right|$ is the total solutions in $\Omega$ set. A value of 0 indicates that all members of Pareto optimal solution are equally spaced. The smaller the value, the better distribution and diversity of non-dominated solutions. Table 6 shows a comparison of the Spacing metric for different algorithms.

**Table 6. Comparison of Spacing metric for different algorithm**

|  | MOIWCA | MODE | MOPSO | NSGA-II |
| --- | --- | --- | --- | --- |
| Best | 0.4336 | 0.4453 | 0.4627 | 0.4852 |
| Worst | 0.6132 | 0.7025 | 0.9416 | 0.9653 |
| Average | 0.5235 | 0.5431 | 0.7123 | 0.7356 |
| Standard Deviation | 0.0397 | 0.0586 | 0.1542 | 0.1653 |

(2) Hyper-volume (HV): This indicator calculates the volume (in the objective space) covered by members of a non-dominated set of solutions $\Omega$ to a problem that works to minimize all objectives. A hypercube $v_{i}$is constructed for each solution $X_{i}\in\Omega$, with reference point *W* and the solution *X_i_* as the diagonal corners of the hypercube. The reference point may be found simply by constructing a vector of the worst objective function values. Thereafter, a union of all hypercubes is found, with the HV of this union calculated as:

$HV=\bigcup_{i=1}^{\left| \Omega\right|} v_{i}$	(41)

Algorithms with larger HV values are desirable. The HV value of a set of solutions is normalized using a reference set of Pareto optimal solutions with the same reference point. After normalization, the HV values are confined to range [0,1]. Table 7 lists the results for each of the four compared algorithms in terms of HV. From Table 7, we can see that the proposed model gets the highest HV values. This means that the MOIWCA has better convergence and diversity performance than the other algorithms.

**Table 7. Comparison of HV metric for different algorithm**

|  | MOIWCA | MODE | MOPSO | NSGA-II |
| --- | --- | --- | --- | --- |
| Best | 0.9486 | 0.8965 | 0.9284 | 0.8652 |
| Worst | 0.7164 | 0.6932 | 0.4683 | 0.2473 |
| Average | 0.8207 | 0.7648 | 0.7136 | 0.5241 |
| Standard Deviation | 0.0519 | 0.0546 | 0.1483 | 0.2062 |

13. It is recommended to increase the understanding of Figs 5 and 9.

**Response**

Thank you for your suggestion. As suggested by reviewer, For Fig 5, we have added our understanding of the figure **(Page 16, lines 318-320)**. The details are as follows:

Fig. 5 shows that *SD_i_*, *BD_i_*, *LD_i_* are the shortest duration, best duration, longest duration of activity *i*, and *BD_i_* corresponds to the maximum value of *QPI*. The parameters can be provided by the deterministic data from the case study.

**Fig.5.** The curve of the relationship of time- quality

For Fig. 9, we have improved the analysis of the Fig 9 **(Pages 29-30, lines 581-592)**. The details are as follows:

Fig 9 shows the typical Pareto optimal fronts obtained using the MOIWCA for this case study. These three fronts clearly show the relationships among project duration, cost, and quality. It is not difficult to see that the relationship between time, cost and quality is non-linear. For example, the project manager can appropriately extend the time and reduce the cost according to the actual situation of the construction project, and the quality can meet the requirements of the construction project; Similarly, the quality can also meet the requirements of the construction project by appropriately reducing the time but increasing the cost. However, the quality of the construction project cannot meet the minimum requirements if the cost is constantly increased by blindly seeking to reduce the time of the construction project or blindly increasing the time to reduce the cost. This three-dimensional representation of the tradeoffs may help decision-makers figure out how the different possible plans for using resources will affect the performance of the project.

**Fig. 9.** Time–cost–quality tradeoff Pareto front using MOIWCA.

14. The figures before equations 7-10 are not clear.

**Response**

Thank you for your suggestion. As suggested by reviewer, we adjusted the size of the figures in equations 7-10, we hope it can meet the reviewer’s standard **(Page 10-11, lines 216, 217, 222 and 223)**.

$s_{j}^{E}-s_{i}^{E}\geq{SS}_{ij}^{min}$	(7)

$f_{k}^{L}-s_{j}^{E}\geq\left( {dur}_{k}-\omega t_{k} \right)+{SS}_{jk}^{min}$	(8)

$f_{j}^{L}-s_{i}^{E}\geq\left( {dur}_{i}-\omega t_{i} \right)+{FF}_{ij}^{min}$	(9)

$f_{k}^{L}-f_{j}^{L}\geq{FF}_{jk}^{min}$	(10)

15. The nomenclature table is recommended for the parameters used in this study.

**Response**

Thank you for your suggestion. As suggested by reviewer, we modify the Table 3, and the new Table 3 is shown as follow **(Page 28, line 559)**.

**Table 3.** Best MOIWCA parameter for 14 activities in the Beijing-Shanghai high speed railway project.

| Parameters | Significance | Value |
| --- | --- | --- |
| *ND* | Population size | 112 |
| *T* | Number of generations | 300 |
| *P_c_* | Crossover probability | 0.85 |
| *P_m_* | Mutation probability | 0.055 |
| *μ* | Expected reproduction index | 0.95 |
| *step_a_* | Wandering step | 0.9 |
| *step_b_* | Calling step | 0.6 |
| *step_c_* | Siege step | 0.5 |
| $\lambda$ | Constant of expected reproduction | 0.3 |

We tried our best to improve the manuscript and made some changes in the manuscript. These changes will not influence the content and framework of the paper. And here we did not list the changes but marked in red in revised paper. We appreciate for Editors/Reviewers’ warm work earnestly, and hope that the correction will meet with approval. Once again, thank you very much for your comments and suggestions.

Sincerely,

Li Xuemei
