# Supplementary material for: Multiple objective immune wolf colony algorithm for solving time-cost-quality trade-off problem
Source: PLoS One. 2023 Feb 9;18(2):e0278634. doi: 10.1371/journal.pone.0278634 (PMC9910742; doi:10.1371/journal.pone.0278634)
Supplement: Attachment — Submitted filename: Response to Reviewers.docx [file pone.0278634.s003.docx]

**Changes made to the Paper**

**Dear Editor**,

The authors gratefully acknowledge the constructive comments on the paper entitled “**Multiple objective immune wolf colony algorithm for solving time-cost-quality trade-off problem**” (Manuscript Number: PONE-D-22-22367R1), offered by the editor and the anonymous referees. We have reviewed the comments and generally agree with those. Accordingly, we have made changes in the paper in line with the referee’s observations to the extent practicable. We take this opportunity to thank each of the individuals involved in the process. We express our sincere gratitude for their in-depth reviews which have helped improve the quality of the paper significantly.

Please find below our response to the comments in the order in which they have been raised.

**Reviewer #1:**

**Comment to the Authors**

Thank you for your consideration.

**Reviewer #2:**

**Comment to the Authors**

1. It is recommended to experimentally report the weak points of the immune wolf colony algorithm. then clarify how the proposed algorithm can overcome these weak points to solve the time-cost-quality trade-off problem.

**Response**

Thank you for your suggestion. As suggested by reviewer, we add the weak of the IWCA, and explain how the MOIWCA perform the TCQT problem in the manuscript **(Pages 26-27, lines 507-529)**. The details are as follows:

In the above content, based on grey wolf optimizer (GWO) algorithm [41] and immune mechanism, we proposed immune wolf colony algorithm (IWCA). It can find the best and unique solution when dealing with single objective problems, but IWCA cannot solve multi-objective optimization problems. The reason is due to the sub objectives of the multi-objective optimization problem are contradictory, and the improvement of one sub objective may cause the performance of another or several other sub objectives to change accordingly (such as the TCQP problem to be solved in this paper). That is, it is impossible to achieve the optimal value of multiple sub objectives at the same time, but only to coordinate and compromise among them, make each sub goal as optimal as possible. Its essential difference from the single objective optimization problem is that the solutions of the multi-objective optimization problem are not unique, but there is a set of optimal solutions composed of many Pareto optimal solutions. Each element in the set is called Pareto optimal solution or non-inferior optimal solution. In order to preform multi-objective optimization problems by IWCA, we combine the idea of multi-objective optimization with the excellent strategy of IWCA, and propose a multi-objective immune wolf colony algorithm (MOIWCA). We learn from the components used in MOPSO [42] and MOGWO [43], and integrate two new components on the basis of IWCA. The first component is to create an archive, which is responsible for storing all non-dominated Pareto optimal solutions generated during algorithm iteration. When the optimal solution is to be archived or the archive is full, it can be controlled through the archive controller. The second component is the leader selection strategy, which is used to select from files *α* wolf, *β* wolves, and *γ* Wolves is the leader in the hunting process. This component can select the least crowded part of the search space in the best archive, and provide one of the non-dominant solutions. The selection is done by roulette-roulette method. These two components have been described in detail in the literature [43], and we will not repeat in the paper.

2. Thank the authors for reviewing more literature but the limitations of already-existing metaheuristic algorithms are not addressed, nor is explained the need to develop an enhanced variant of an optimization algorithm. Thus, more new and improved metaheuristic algorithms such as the following studies should be considered. Starling murmuration optimizer: A novel bio-inspired algorithm for global and engineering optimization, QANA: Quantum-based avian navigation optimizer algorithm, DMDE: Diversity-maintained multi-trial vector differential evolution algorithm for non-decomposition large-scale global optimization.

**Response**

Thank you for your suggestions. we have added these papers in the manuscript **(Page 5, lines 102)**. Our reply is as follows:

At present, common optimization algorithms include particle swarm optimization algorithm, fast non-dominated sorting genetic algorithm with elite retention strategy (NSGA-II), ant colony algorithm, simulated annealing algorithm, and so forth [22-24].

The references (22-24) are advised by reviewer.

3. It is recommended to make sense of results gained by different algorithms and their differences for the spacing metrics using Eq. (40).

**Response**

Thank you for your suggestions. As suggested by reviewer, we have revised in the manuscript **(Page 34, lines 663-667)**. Our reply is as follows:

The smaller the value of the Spacing metric, the better distribution and diversity of non-dominated solutions. From the comparison results of the Spacing metric for different algorithms, the MOIWCA obtained the minimum Spacing metric. This shows that in dealing with multi-objective optimization problems, The non-dominated solutions obtained by MOIWCA have better distribution and diversity than other algorithms.

4. It is recommended to boost the visualization of this study by increasing the experimental evaluations.

**Response**

Thank you for your suggestions. In the paper, we add Fig 13 and Fig 14 to improve the visualization of this paper.

**Fig 13. Comparison of Spacing metric for different algorithm.**

**Fig 14. Comparison of HV metric for different algorithm.**

5. The conclusion section also needs significant revisions. It should be linked to research questions, and address the research contributions.

**Response**

Thank you for your suggestions. As suggested by reviewer, we revise the conclusion in the manuscript **(Page 39, lines 689-705).** Our reply is as follows:

In this paper, the time-cost-quality trade-off problem is studied, and a hybrid algorithm MOIWCA is developed to solve the TCQT problem for construction project. The paper makes three important contributions: first, based on the traditional time-cost model, we introduce the bonus-penalty mechanism, and propose a new nonlinear time-cost model. Meanwhile, we develop a new QPI model by analyzing the qualitative and quantitative relationship between time and quality. Second, we propose a multi-objective immune wolf colony algorithm (MOIWCA)to solve the TCOQ problem. The design of algorithm is divided into two parts: we propose IWCA that combines the wolf colony algorithm and immune algorithm, and improves the crossover operation and mutation operation. In addition, we integrate IWCA with multi-objective optimization to enable it to deal with time-cost-quality trade-off problem. Thirdly, we take the Beijing-Shanghai high-speed railway construction project as a case to evaluate the performance of the MOIWCA. The results show that the MOIWCA is more effective and efficient than widely used multi-objective algorithms (MOPSO, NSGA-II, MODE). The SP and HV values comparisons indicated that the proposed MOIWCA performs excellent in terms of distribution, convergence and uniformity.

Results show that the proposed method MOIWCA generates a better Pareto front than widely used approaches. With the obtained non-dominated solutions, the project managers can easily trade-off the three important objectives, including project time, cost, and quality.

We tried our best to improve the manuscript and made some changes in the manuscript. These changes will not influence the content and framework of the paper. And here we did not list the changes but marked in red in revised paper. We appreciate for Editors/Reviewers’ warm work earnestly, and hope that the correction will meet with approval. Once again, thank you very much for your comments and suggestions.

Sincerely,

Li Xuemei
